# Low attainment to PK/PD-targets for β-lactams in a multi-center study on the first 72 h of treatment in ICU patients

**DOI:** 10.1038/s41598-022-25967-9

**Published:** 2022-12-19

**Authors:** Anna-Karin Smekal, Mia Furebring, Erik Eliasson, Miklos Lipcsey

**Affiliations:** 1 Department of Surgical Sciences, Anaesthesiology and Intensive Care, Uppsala University, Uppsala, Sweden; 2 Clinical Microbiology, Karolinska University Hospital, Solna, Stockholm, Sweden; 3 Department of Medical Sciences, Section of Infectious Diseases, Uppsala University, Uppsala, Sweden; 4 Clinical Pharmacology at Laboratory Medicine and Medical Diagnostics MDK/KUL, Karolinska Institutet and Karolinska University Hospital, Stockholm, Sweden; 5 Department of Surgical Sciences, Hedenstierna Laboratory, Uppsala University, Uppsala, Sweden

**Keywords:** Antimicrobial therapy, Bacterial infection, Medical research

## Abstract

Severe infections are life-threatening conditions commonly seen in the intensive care units (ICUs). Antibiotic treatment with adequate concentrations is of great importance during the first days when the bacterial load is the highest. Therapeutic drug monitoring (TDM) of β-lactam antibiotics has been suggested to monitor target attainment and to improve the outcome. This prospective multi-center study in seven ICUs in Sweden investigated pharmacokinetic/pharmacodynamic-target (PK/PD-target) attainment for cefotaxime, piperacillin-tazobactam and meropenem, commonly used β-lactams in Sweden. A mid-dose and trough antibiotic concentration blood sample were taken from patients with severe infection daily during the first 72 h of treatment. Antibiotic plasma concentrations were analysed by liquid chromatography-mass spectrometry (LC–MS). Antibiotic concentrations 100% time above MIC (minimal inhibitory concentration), (100% T > MIC) and four times above MIC 50% of the time (50% T > 4xMIC) were used as PK/PD-targets. We included 138 patients with the median age of 67 years and the median Simplified Acute Physiology Score 3 (SAPS3) of 59. Forty-five percent of the study-population failed to reach 100% T > MIC during the first day of treatment. The results were similar the following two days. There was a three-fold risk of not meeting the PK/PD target if the patient was treated with cefotaxime. For the cefotaxime treated patients 8 out of 55 (15%) had at least one end-dose concentrations below the level of detection during the study. Low age, low illness severity, low plasma creatinine, lower respiratory tract infection and cefotaxime treatment were risk factors for not reaching 100% T > MIC. In Swedish ICU-patients treated with β-lactam antibiotics, a high proportion of patients did not reach the PK/PD target. TDM could identify patients that need individual higher dosing regimens already on the first day of treatment. Further studies on optimal empirical start dosing of β-lactams, especially for cefotaxime, in the ICU are needed.

**Trial registration: **The protocol was retrospectively registered 100216 (ACTRN12616000167460).

## Introduction

Severe infections are the most common cause of emergency admission to intensive care units (ICUs) globally^1^ and almost 70% of patients in the ICU are treated with antibiotics at some point^2^. Early treatment with efficient antibiotics has been proven to be lifesaving in septic shock^3–6^. Despite this, the mortality and morbidity caused by infections in ICUs remains high. One reason could be that treatment with β-lactams in the ICU patient often result in suboptimal antibiotic concentrations^7–10^. Standard antibiotic dosing is often adopted from pharmacokinetic studies in healthy volunteers and non-critically ill patients. However, pharmacokinetics in critically ill patients is altered with great inter- and intra-variation in total body water, plasma protein levels, volume of distribution, renal and liver function compared to healthy volunteers^11–13^. The altered pharmacokinetics of β-lactams in ICU patients increases the risk of underdosing with potential impact on outcome and the development of antimicrobial resistance^14^. Moreover, it also increases the risk of overdosing with possible toxic side effects.

The bacterial load in sepsis and septic shock is highest when the first doses of antibiotics are administered^15,16^, thus attaining adequate antibiotic concentrations during the first days of treatment in the ICU is of great importance in order to prevent mortality and morbidity.

Several studies have reported the serum antibiotic concentrations in ICU patients^7–9^, but they did not study the first critical days of antibiotic treatment. Some of them also included a large proportion of patients on antibiotic prophylaxis rather than active treatment of infections.

In a recently published position paper article with guidelines for therapeutic drug monitoring (TDM) from European experts of among others ESICM (European Society of Intensive Care Medicine) and ESCMID (European Society of Clinical Microbiology and Infectious Diseases), concentration sampling 24–48 h after antibiotic initiation are recommended for β-lactams in critically ill patients^17^. However, we hypothesised that an earlier start of sampling could be beneficial for ICU-patients.

Accordingly, we performed a prospective multi-center observational study in seven Swedish ICUs comparing the minimal inhibitory concentration (MIC) that the initial empiric treatment should cover, with the concentration at the end of dosing interval and in the middle of the dosing interval, giving a dichotomous outcome for target attainment for 100% T > MIC and 50% T > 4xMIC.

The primary objective was to assess if antibiotic concentrations in serum met the pharmacokinetic/pharmacodynamics (PK/PD) targets during the first 72 h of antibiotic treatment. The secondary objectives were to assess the association between reaching the PK/PD target versus patient and treatment characteristics.

## Material and methods

### Ethical approval

The study was approved by the regional ethics review board in Uppsala (No. 2015/135). Informed consent was obtained from the patient or next of kin if the patient was unable give consent. The Declaration of Helsinki and its subsequent revisions was followed. STROBE guidelines were used for reporting. The protocol was submitted to a trial registry 100216 (ACTRN12616000167460).

### Study design

The Antibiotic Concentrations in Critical ill ICU-patients in Sweden (ACCIS) study, was a prospective multi-center study, performed between December 2015 and July 2017 in 7 intensive care units (ICUs) of variable size in Sweden, see Supplementary information, (Additional file 1).

### Patients

Patients above 18 years of age with a severe suspected or confirmed infection were eligible for inclusion if intravenous antibiotic treatment were initialized no longer than 24 h prior to inclusion in the study. Only patients that were given the most common used antibiotics in Swedish ICUs could be included. For each patient the study continued for a maximum of three days. Exclusion criteria were known pregnancy, intermittent hemodialysis or limitations of care. Patients could only be included once in the study. Antibiotic therapy was prescribed by the attending physician and/or the infectious disease (ID) consultant at the unit. The study investigators had no influence on the therapeutic strategy or dosing.

### Clinical data collection

Demographic data, and daily fluid balance, daily laboratory variables, time on kidney replacement therapy (KRT), doses of vasopressor treatment, doses and duration of antibiotic treatment, concomitant medication and suspected infection were registered in a study database. Patients were followed up to 30 days after inclusion for mortality and final diagnosis of infection.

### Handling of antibiotic concentration samples

Two blood samples a day were collected in serum tubes during the three study days. One was taken between two doses (mid-dose) and one immediately before the next antibiotic dose (end of the dosing interval). Each sample was centrifuged for 7 min at 2400 *g*, transported to Nunc CryoTubes and stored at − 70 to − 80 degrees within 30 min of sampling.

Plasma concentrations of cefotaxime, piperacillin and meropenem were determined by an established method based on liquid chromatography combined with tandem mass spectrometry (LC-MS/MS) at the SWEDAC-quality assured hospital laboratory in the Department of Clinical Pharmacology, Karolinska University Hospital Huddinge, essentially as described before^9^. In short, after addition of deuterated internal standards, plasma samples were subject to protein precipitation followed by injection using a Thermo PAL autosampler onto the TSQ Quantum Ultra LC–MS/MS system. The quantification range was 0.50–50 µg/mL, 0.20–100 µg/mL and 0.20–50 µg/mL for cefotaxime, piperacillin and meropenem respectively. Tazobactam levels were not measured.

### Treatment targets

A plasma antibiotic concentration higher than the MIC at trough (100% T > MIC)^17^ or a concentration 4 times higher than the MIC at mid-dose (50% T > 4xMIC)^7^ were considered as achieved PK/PD treatment target.

In the target attainment calculations, we used the worst-case scenario MIC (MIC_WCS_) for each β-lactam, which reflects the highest MIC the initial empiric treatment should cover. For cefotaxime the epidemiological cut-off value for *S. aureus* of 4 mg/L was used^18^. For piperacillin-tazobactam and meropenem the clinical MIC-breakpoint for *P. aeruginosa* of 16 mg/L respectively 2 mg/L were used^19,20^.

Overdosing was defined as concentrations above threshold of toxicity for piperacillin-tazobactam and meropenem as suggested by European experts^17^. There is no established toxicity threshold for cefotaxime probably due to its low ability to cause neuro- or nephrotoxicity compared to other cephalosporins^21^.

### Point prevalence measurement

Two point-prevalence assessments of which antibiotics used for treatment of infection were conducted in the seven participating ICUs on two different days, with two months apart.

### Statistical analysis

To be able to show that 10% of the patients did not attain PK/PD targets, at least 16 patients had to be included for each studied antibiotics for an alpha error of 0.05 and beta error of 0.8. We aimed at including 150 patients i.e. 10% more than suggested above.

Missing antibiotic concentrations were imputed using Multivariate Imputation by Chained Equations (MICE, R software version 3.5.3).

Data were presented as median (IQR) or as number of observations (%) unless otherwise stated. Spearman Rank Order Correlations were used to assess associations between continuous variables. Univariate logistic regression was performed with 100% T > MIC to assess risk factors of not reaching treatment targets. STATISTICA software, version 13.2 (StatSoft, Tulsa, OK) was used for calculations.

## Results

### Patients

**Figure 1:**
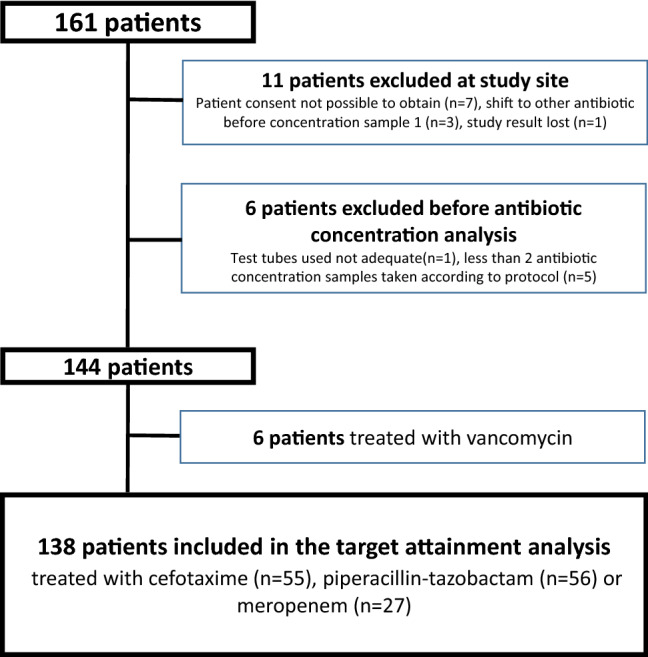
Flow chart illustrating the patients in the study.

One hundred forty-four patients were included of whom 55 were treated with cefotaxime, 56 with piperacillin-tazobactam, 27 with meropenem and 6 patients treated with vancomycin (Fig. 1).

During the two days of point prevalence assessment 44% (31/71) of the ICU patients received antibiotics as treatment for an infection. The proportion of patients receiving cefotaxime, piperacillin-tazobactam, meropenem or vancomycin was 72%.

Patient characteristics are presented in Table 1. Community- acquired infections (symptoms developed before hospitalization or within 48 h after admission) were seen in 52% (75/144) of the patients and the rest had a hospital-acquired infection (symptoms developed more than 48 h after admission to hospital). Lower respiratory tract infections (LRTI) were most common followed by intra-abdominal infections (IAI) and skin and soft tissue infections (SSTI).

**Table 1:** Demographic and clinical characteristics of the included patients. Data are presented as median (IQR) or number (percentages).

Characteristic	Cefotaxime (n = 55)	Piperacillin-tazobactam (n = 56)	Meropenem (n = 27)	Vancomycin (n = 6)	Total (n = 144)
Age, year	64 (47–73)	68 (54–76)	67 (57–73)	70 (64–76)	67 (55–74)
Male gender	37 (67%)	34 (61%)	13 (48%)	5 (83%)	89 (62%)
Body weight, kg	92 (79–101)	80 (66–97)	83 (70–99)	87.7 (80–100)	85 (73–100)
SAPS3	55 (47–64)	63.5 (52–74.5)	62 (50–69)	78 (52–86)	59 (50–69)
Vasopressor treatment	37 (67%)	42 (75%)	23 (85%)	5 (83%)	107 (74%)
KRT	4 (7%)	12 (21%)	9 (33%)	3 (59%)	28 (19%)
Immunosuppression	1 (2%)	7 (13%)	6 (22%)	1 (16%)	15 (10%)
30-day mortality	4 (7%)	19 (18%)	9 (33%)	3 (50%)	26 (18%)
Hospital-acquired infection	19 (35%)	32 (57%)	13 (48%)	5 (83%)	69 (48%)
Community-acquired infection	36 (65%)	24 (42%)	14 (52%)	1 (17%)	75 (52%)
**Focus of infection**					
LRTI	30 (55%)	25 (45%)	10 (37%)	2 (33%)	67 (47%)
IAI	1 (2%)	16 (29%)	4 (15%)	2 (33%)	23 (16%)
SSTI	16 (29%)	2 (4%)	5 (19%)	1 (17%)	24 (17%)
UTI	4 (7%)	6 (11%)	3 (11%)	–	13 (9%)
Other	4 (7%)	7 (13%)	5 (19%)	1 (17%)	17 (27%)
Blood culture before start of antibiotic treatment	51 (92%)	52 (92%)	26 (96%)	5 (83%)	134 (93%)
Plasma creatinine concentration (µmol/L)	92 (68–155)	128 (79–187)	130 (71–190)	102 (69–299)	118 (71–185)
Plasma albumin (g/L)	23 (16–29)	26 (22–29)	24 (22–27)	21 (15–27)	24 (20–29)
Blood hemoglobin (g/L)	118 (99–131)	105 (93–121)	98 (90–108)	92 (88–98)	108 (95–124)
Daily dose (g/24 h) In day 1, 2 and 3 order	4.0 (3.0–6.0)	12 (12.0–16.0)	3.0 (1.5–4.0)	3.0 (2.0–3.0)	NA
	3.0 (3.0–6.0)	12 (12.0–12.0)	2.8 (1.5–3.0)	2.5 (2.0–3.0)	
	3.0 (3.0–6.0)	12 (12.0–12.0)	3.0 (1.5–3.0)	2.0 (1.0–3.0)	
Antibiotic concentration (mg/L) Mid dosing interval In day 1, 2 and 3 order	7.8 (4.0–15.0)	67.0 (38.0–99)	13.8 (5.2–18.8)	NA	NA
	8.5 (4.3–13.4)	64.1 (31.9–103)	12.7 (6.5–17.8)		
	8.6 (4.1–15.5)	62.6 (36.4–112)	7.7 (4.8–14.5)		
Antibiotic concentration (mg/L) End dosing interval In day 1, 2 and 3 order	2.89 (1.25–6.85)	26.7 (7.5–57.7)	4.3 (1.8–9.5)	NA	NA
	2.45 (1.15–5.11)	30.0 (7.7–60.4	4.9 (2.4–8.1)		
	2.22 (0.93–5.08)	19.5 (9.1–68.8)	2.6 (1.2–6.7)		

*KRT* kidney replacement therapy.

Immunosuppression: Neutropenic fever, stem cell-/solid organ transplantation OR prednisolone treatment of more than 20 mg per day due to inflammatory rheumatic disease or haematological diseases at admission.

Hospital-acquired infection: Symptoms developed more than 48 h after admission to hospital.

Community-acquired infection: Symptoms developed before hospitalisation or within 48 h after admission.

Focus of infection: *LRTI* lower respiratory tract infection, *IAI* intra-abdominal infection, *SSTI* soft skin and tissue infection, *UTI* urinary tract infection.

Only the 138 patients treated with the β-lactams were included in the target attainment analysis part of the study since the six vancomycin treated patients were considered too few to analyse further.

### Antibiotic concentrations

Cefotaxime and meropenem were given as intermittent injections. Piperacillin-tazobactam was given as an injection in 79% (44/56) of the patients, while the remaining patients received a 30 min infusion. No initial higher loading doses were given in the study.

Sixty-nine percent of the patients (95/138) were included in the study prior to the first dose of antibiotics, 23% (32/138) on the second dose.

The antibiotic concentrations at mid-dosing interval and end-dosing interval are reported in Table 1. In 8 of 55 patients (15%) treated with cefotaxime at least one end-dose concentration was below the lower level of detection (LLOD) of < 0.5 mg/L. Of these patients, 25% (2/8) had undetectable end-dosing levels all three days and for the rest the concentrations were below 1.25 mg/L during the three study days. Twenty-one of 55 patients (38%) treated with cefotaxime had at least one end dosing concentration below 1 mg/L.

None of the patients treated with piperacillin-tazobactam or meropenem had end-dose antibiotic concentrations below LLOD or above the threshold of toxicity.

The correlation between antibiotic concentrations day 1 vs. 2 and day 1 vs. 3 were high, see Supplementary Information, (Additional file 2, Table S1). For the concentrations in the KRT group see Supplementary information, (Additional file 3, Table S2).

### PK/PD targets attainments for cefotaxime, piperacillin and meropenem

**Figure 2:**
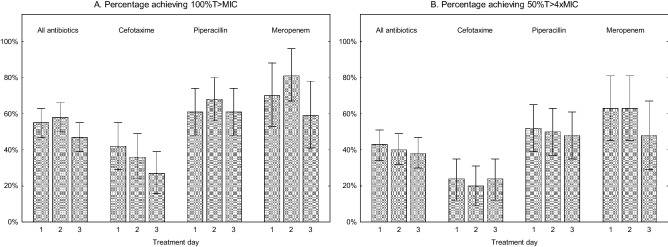
(**A**) Percentage achieved 100% T > MIC. (**B**) Percentage achieved 50% T > 4xMIC.

In the whole cohort, 62 of 138 (45%) patients did not reach 100% T > MIC on the first day of treatment. The results were similar for the following days (Fig. 2a). When using 50% T > 4xMIC the results were even lower (Fig. 2b). There was no trend towards better target attainment results day 3 compared to day 1 regardless of which PK/PD-targets that was used.

For cefotaxime 58% (32/55) of the patients did not reach 100% T > MIC the first day (Fig. 2a). The corresponding results for piperacillin-tazobactam were 39% (22/56) not reaching 100% T > MIC (Fig. 2a) and for meropenem 30% (8/27) (Fig. 2a). The results for day 2 and 3 were comparable to day 1. Target attainment was lower when 50% T > 4xMIC was used as the PK/PD target, in particular in cefotaxime treated patients where almost 80% did not meet that target (Fig. 2b).

### Risk factors for low or high target attainment

**Figure 3:**
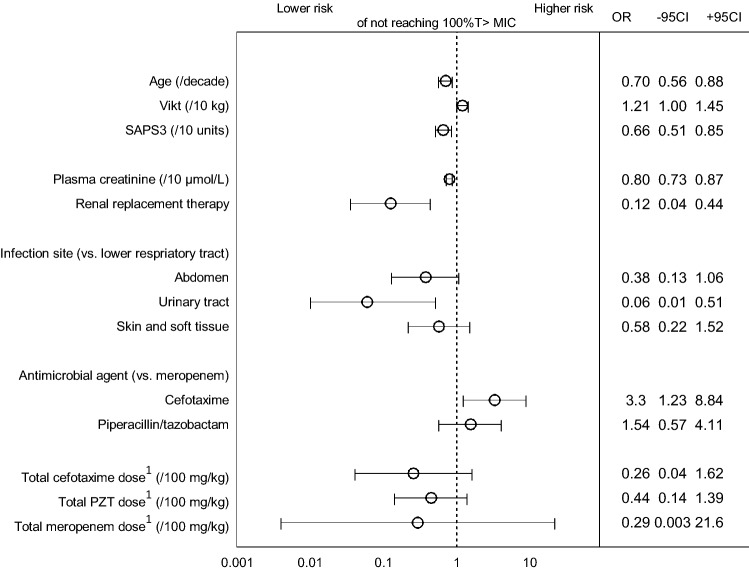
Forest plot illustrating predictive factors resulting in lower (to the left) or higher (to the right) risk for target failure of not reaching 100% T > MIC including odds ratios (OR) with 95% confidence intervals from univariate logistic regression. ^1^Daily doses of antibiotics per body weight.

Increasing age, SAPS3 and plasma creatinine were associated with reaching target attainment of 100% T > MIC (Fig. 3)**,** and KRT was associated with an eight-fold decreased risk of target failure. There was a 16-fold lower risk for reaching 100% T > MIC in patients with LRTI compared with patients with UTI.

In patients treated with cefotaxime there was a three-fold risk of not reaching 100% T > MIC. The total daily dose/kg was not associated with 100% T > MIC. However, variation in dosing was limited, thus finding an effect of different dosing regimens was less likely (Table 1).

## Discussion

In Swedish ICU patients treated for infection with cefotaxime, piperacillin-tazobactam or meropenem, we found that 45% did not reach the PK/PD target 100% T > MIC during the important first days of treatment. The failure was even greater using the target 50% T > 4xMIC. No patient had toxic levels of these antibiotics. Low age, low illness severity, low creatinine, LRTI and cefotaxime treatment were risk factors for not reaching 100% T > MIC.

Target failure in 45% of patients in our study is comparable with previous reports on β-lactam antibiotics in ICU patients reporting 40–45% of the patients not reaching the PK/PD target^7,8,10^. However, we purposely left out the protein binding in our calculations, in order to see what the best possible outcome could be in our ICU-population and despite this, the target attainment is this low.

We report that end interval sampling during the first day after antibiotic initiation often can identify patients with risk of very low antibiotic concentrations on the following days that will need higher dosing regimen, especially for cefotaxime treated patients. This is in contrast with current European recommendations that TDM can be started first after 48 h of antibiotic treatment with β-lactams due to steady-state^17^.

In our study 15% of the patients had at least one end-dose concentration of cefotaxime below detection limit and a low concentration on day 1 was likely to continue the following days. These findings and the fact that no patient reached toxic concentrations of the studied antibiotics opens for discussion of changes in standard dosing regimens for β-lactams in ICU-patients, especially for cefotaxime and piperacillin-tazobactam.

Younger age with increased renal clearance were associated with target attainment failure whereas a high SAPS3 and a high age were associated with target attainment. This is in line with previous studies and stresses that TDM in young patients with augmented renal function is important^8,17,22^. Conversely, patients on KRT have a greatly decreased risk of not attaining treatment targets. In the KRT population piperacillin/tazobactam and meropenem were the most common agents.

Despite cases where high antibiotic doses, such as cefotaxime of 2 g 3 times daily, were given target attainment was not reached in many cases. Moreover, we did not see association between the antibiotic dosing and target attainment. However, confidence intervals of antibiotic dosing were wide in the logistic regression model, probably due to low variation in dosing per body weight. For all three antibiotics there was a trend for not reaching treatment targets with lower doses. Additionally, the attending physician/ID consultant prescribing antibiotics may have adjusted the antibiotic dose to clinical factors influencing the pharmacokinetics, thus facilitating target attainment.

### Strengths and limitations

To our knowledge, the ACCIS study is the first multi-center study to study the PK/PD-target attainment during the first three critical days after start of antibiotic treatment with cefotaxime, piperacillin-tazobactam, or meropenem in ICU-patients treated for an infection.

However, the study has some limitations. Firstly, antibiotic concentration levels in the study are total antibiotic concentrations and not the free fraction in serum. In earlier studies, the free fraction was estimated from protein binding data in healthy volunteers^23^. However, this might not be appropriate in the ICU-setting since protein levels and binding in ICU-patients are altered^24,25^. Accordingly, protein binding was not included in our calculations in order to see what the best possible outcome regarding target attainment could be.

Secondly, as in other PK/PD-studies in ICU patients, we measured antibiotic concentrations in the bloodstream and not at the site of infection. Moreover, the antibiotic concentrations were measured on the first day probably before steady-state in plasma was established, thereby reducing the predictive value of a single TDM-value over the coming days. However, in the critically ill with unstable pharmacokinetics steady state might never be achieved. In our study, low antibiotic concentration for cefotaxime from day 1 were reproducible on day 2 and 3 indicating that early TDM indeed can provide useful information. As in many previous studies we used the MIC value of the worst case scenario bacteria the empirical therapy should cover instead of the actual MIC of the causative bacteria. It is possible that this could have influenced our target attainment results^8^.

Another limitation of the study is that data are from 2015 and 2017 and based on intermittent injections of the beta-lactam antibiotics without an initial higher bolus dose. Presently, it remains controversial whether continuous or prolonged infusion is of benefit in critically ill patients. The data from this study provides further support that the use of intermittent injections of beta-lactams leads to failure of reaching target attainment during the first days of treatment. One way of mitigating this could be optimization of the dosing through prolonged infusion*.*

### Future studies

This study supports the need of studies on optimized empirical standard start dosing regimens for the β-lactam treated patients in the ICU. After characterisation of critical co-variates, there is a great potential to use pharmacometrics-based models in the prediction of individual starting dose and adequate dose adjustments based on TDM. Also the impact on different MIC-parameters on target attainment calculations needs further investigations. Here, analysis of protein-unbound concentrations of beta-lactam antibiotics in plasma may be required to better understand the PK/PD-relationships in vivo.

## Conclusion

In Swedish ICU patients treated with β-lactam antibiotics, a high proportion of patients did not reach the PK/PD target of 100% T > MIC. TDM already on the first day of treatment could identify patients that need higher individual dosing regimens.

## Supplementary Information

Supplementary Information.

## Data Availability

Data are available from the corresponding author on reasonable request.

## Abbreviations

ACCIS: Antibiotic concentrations in ill ICU-patients in Sweden
ICU: Intensive care unit
ID consultant: Infectious disease consultant
LC–MS: Liquid chromatography-mass spectrometry
LLOD: Lower level of detection
LRTI: Lower respiratory tract infections
MIC: Minimal inhibitory concentration
PK/PD: Pharmacokinetics/Pharmacodynamics
KRT: Kidney replacement therapy
SAPS3: Simplified acute physiology score 3
SSTI: Skin and soft tissue infections
TDM: Therapeutic drug monitoring
UTI: Urinary tract infection

## References

[1] Perner A (2016). Sepsis: Frontiers in diagnosis, resuscitation and antibiotic therapy. Intensive Care Med..

[2] Vincent JL (2009). International study of the prevalence and outcomes of infection in intensive care units. JAMA.

[3] Evans L (2021). Surviving sepsis campaign: International guidelines for management of sepsis and septic shock 2021. Intensive Care Med..

[4] Seymour CW (2017). Time to treatment and mortality during mandated emergency care for sepsis. N. Engl. J. Med..

[5] Liu VX (2017). The timing of early antibiotics and hospital mortality in sepsis. Am. J. Respir. Crit. Care Med..

[6] Ferrer R (2014). Empiric antibiotic treatment reduces mortality in severe sepsis and septic shock from the first hour: Results from a guideline-based performance improvement program. Crit. Care Med..

[7] Roberts JA (2014). DALI: Defining antibiotic levels in intensive care unit patients: Are current beta-lactam antibiotic doses sufficient for critically ill patients?. Clin. Infect. Dis..

[8] Woksepp H (2017). High target attainment for beta-lactam antibiotics in intensive care unit patients when actual minimum inhibitory concentrations are applied. Eur. J. Clin. Microbiol. Infect. Dis..

[9] Petersson J, Giske CG, Eliasson E (2016). Standard dosing of piperacillin and meropenem fail to achieve adequate plasma concentrations in ICU patients. Acta Anaesthesiol. Scand..

[10] Taccone FS (2010). Insufficient beta-lactam concentrations in the early phase of severe sepsis and septic shock. Crit. Care.

[11] Blot SI, Pea F, Lipman J (2014). The effect of pathophysiology on pharmacokinetics in the critically ill patient–concepts appraised by the example of antimicrobial agents. Adv. Drug Deliv. Rev..

[12] Zander J (2016). Piperacillin concentration in relation to therapeutic range in critically ill patients–a prospective observational study. Crit. Care.

[13] Roberts JA (2014). Individualised antibiotic dosing for patients who are critically ill: Challenges and potential solutions. Lancet Infect. Dis..

[14] Sumi CD (2019). What antibiotic exposures are required to suppress the emergence of resistance for gram-negative bacteria? A systematic review. Clin. Pharmacokinet..

[15] Whimbey E (1984). Clinical correlations of serial quantitative blood cultures determined by lysis-centrifugation in patients with persistent septicemia. J. Clin. Microbiol..

[16] Lipcsey M (2018). Should the aminoglycoside β-Lactam combination be abandoned in all severely ill patients with presumed gram-negative infection?. Clin. Infect. Dis..

[17] Abdul-Aziz MH (2020). Antimicrobial therapeutic drug monitoring in critically ill adult patients: A position paper(). Intensive Care Med..

[18] *EUCAST (European committee on antimicrobial susceptibility testing). Cefotaxime MIC distributions. Antimicrobial wild type distributions of microorganisms.* Available from: https://mic.eucast.org/search/?search%5Bmethod%5D=mic&search%5Bantibiotic%5D=38&search%5Bspecies%5D=-1&search%5Bdisk_content%5D=-1&search%5Blimit%5D=50 Accessed 15 May 2022.

[19] *EUCAST ( European Committee on Antimicrobial Susceptibility Testing). Piperacillin-tazobactam MIC distributions. Antimicrobial wild type distributions.* Available from: https://mic.eucast.org/search/?search%5Bmethod%5D=mic&search%5Bantibiotic%5D=155&search%5Bspecies%5D=-1&search%5Bdisk_content%5D=-1&search%5Blimit%5D=50 Accessed 15 May 2022.

[20] *EUCAST (European Committee on Antimicrobial Susceptibility Testing). Meropenem MIC distributions. Antimicrobial wild type distributions*. Available from: https://mic.eucast.org/search/?search%5Bmethod%5D=mic&search%5Bantibiotic%5D=123&search%5Bspecies%5D=-1&search%5Bdisk_content%5D=-1&search%5Blimit%5D=50 Accessed 15 May 2022.

[21] Doerr BI (1982). Cefotaxime toxicity studies: A review of preclinical studies and some clinical reports. Rev. Infect. Dis..

[22] Huttner A (2015). Therapeutic drug monitoring of the β-lactam antibiotics: What is the evidence and which patients should we be using it for?. J. Antimicrob. Chemother..

[23] Craig WA (1998). Pharmacokinetic/pharmacodynamic parameters: Rationale for antibacterial dosing of mice and men. Clin. Infect. Dis..

[24] Hayashi Y (2010). Pharmacokinetic evaluation of piperacillin-tazobactam. Expert. Opin. Drug Metab. Toxicol..

[25] Liebchen U (2014). Unbound fraction of ertapenem in intensive care unit patients. J. Antimicrob. Chemother..

