# Supplementary material for: Low attainment to PK/PD-targets for β-lactams in a multi-center study on the first 72 h of treatment in ICU patients
Source: Sci Rep. 2022 Dec 19;12:21891. doi: 10.1038/s41598-022-25967-9 (PMC9763385; doi:10.1038/s41598-022-25967-9)
Supplement: Supplementary file 1 — Supplementary Information. [file 41598_2022_25967_MOESM1_ESM.docx]

**Low attainment to PK/PD-targets for β-lactams in a multi-center study on the first 72 hours of treatment in ICU patients**

## Anna-Karin Smekal, Mia Furebring, Erik Eliasson, Miklos Lipcsey

**Additional File 1: Participating ICUs in the study**:

General ICU Uppsala University Hospital, Cardiothoracic ICU Uppsala University Hospital, Burn ICU Uppsala University Hospital, General ICU Gävle County Hospital, General ICU Karlstad County Hospital, General ICU Eskilstuna County Hospital and General ICU Västerås County Hospital.

ICU teams:

Uppsala University Hospital, General ICU, Joanna Wessbergh and Elin Söderman research nurses. David Smekal, ICU physician. Cardiothoracic ICU: Anita Bertilsson, research nurse, Fredrik Lennmyr ICU physician. Burn ICU: Marie Lindblad, research nurse, Fredrik Huss ICU physician.

Gävle County Hospital, General ICU, Magdalena Lundh and Maria Lingman, ICU-nurses. Johan Rasmusson ICU physician.

Karlstad County hospital General ICU: Marie Skålerud and Marianne Hållbus, ICU-nurses. Niklas Kullander and Jon Rosell ICU physicians.

Västerås County Hospital General ICU: Wilhelm Wallqvist, ICU physician. Clinic of infectious diseases: Anne-Marie Bernövall, nurse and Anna Hill, ID physician.

Eskilstuna County Hospital: Andreas Pikwer, ICU physician.

**Additional File 2: Table S1**

Table S1. Correlation coefficients (rho) for antibiotic concentrations day 1 vs 2 and 1 vs 3.

| Correlation | Cefotaxime | Piperacillin-tazobactam | Meropenem |
| --- | --- | --- | --- |
| Mid-dosing interval day 1 and 2 | 0.81 | 0.87 | 0.77 |
| End-dosing interval day 1 and 2 | 0.87 | 0.89 | 0.82 |
| Mid-dosing interval day 1 and 3 | 0.83 | 0.71 | 0.60 |
| End-dosing interval day 1 and 3 | 0.81 | 0.78 | 0.82 |

p<0.001 for all

**Additional File 3: Table S2**

Table S2. Antibiotic concentrations in patients with kidney replacement therapy. Data are presented as median (IQR).

| Characteristic | Cefotaxime (n=4) | Piperacillin-tazobactam (n=12) | Meropenem (n=9) | Total (n=25) |
| --- | --- | --- | --- | --- |
| Antibiotic Concentration (mg/L)  Mid dosing interval  In day 1, 2 and 3 order | 21.2 (8.8-71.9)  22.1 (10.4-30.8)  26.4 (7.3-50.5) | 75.8 (44.2-119)  84.1 (58.3-141.5)  74.6 (48.3-123) | 15.3 (9.5-18.8)  14.4 (12.7-20.3)  12.1 (7.6-21.8) | NA |
| Antibiotic Concentration (mg/L)  End dosing interval  In day 1, 2 and 3 order | 9.3 (4.6-16.7)  9.9 (3.1-16.0)  3.5 (2.9-15.9) | 47.2 (18.8-71.5)  46.65 (33.0-90.6)  30.35 (18.3-79.0) | 7.2 (3.2-12.7)  5.9 (5.3-8.2)  6.5 (4.6-16.9) | NA |
